# Exploration of the lactation function of protein phosphorylation sites in goat mammary tissues by phosphoproteome analysis

**DOI:** 10.1186/s12864-021-07993-5

**Published:** 2021-09-28

**Authors:** Chao Zhu, Junru Zhu, Quyu Duan, Yue Jiang, Hao Yin, Yonglong He, Fu Li, Xiao-Peng An

**Affiliations:** College of Animal Science and Technology, Northwest A&F University, No. 22 Xinong Road, Shaanxi 712100 Yangling, People’s Republic of China

**Keywords:** Phosphoproteome, Lactation, IRS1, β-casein, Triglycerides, Goat

## Abstract

**Background:**

Protein phosphorylation plays an important role in lactation. Differentially modified phosphorylation sites and phosphorylated proteins between peak lactation (PL, 90 days postpartum) and late lactation (LL, 280 days postpartum) were investigated using an integrated approach, namely, liquid chromatography with tandem mass spectrometry (LC-MS/MS) and tandem mass tag (TMT) labeling, to determine the molecular changes in the mammary tissues during the different stages of goat lactation.

**Results:**

A total of 1,938 (1,111 upregulated, 827 downregulated) differentially modified phosphorylation sites of 1,172 proteins were identified (*P* values < 0.05 and fold change of phosphorylation ratios > 1.5). Multiple phosphorylation sites of FASN, ACACA, mTOR, PRKAA, IRS1, RPS6KB, EIF4EBP1, JUN, and TSC2 were different in PL compared with LL. In addition, the Kyoto Encyclopedia of Genes and Genomes (KEGG) enrichment analysis showed that the calcium signaling pathway, oxytocin signaling pathway and MAPK signaling pathway were enriched. The western blot results showed that the phosphorylation levels of ACACA (Ser80), EIF4EBP1 (Thr46) and IRS1 (Ser312) increased and JUN (Ser63) decreased in PL compared with LL. These results were consistent with the phosphoproteome results.

**Conclusions:**

In this study, we identified for the first time the differentially modified phosphorylation sites in goat mammary tissues between PL and LL. These results indicate that the multiple differentially modified phosphorylation sites of FASN, ACACA, mTOR, PRKAA, IRS1, RPS6KB, EIF4EBP1, TSC2, and JUN and proteins involved in the calcium signaling pathway, oxytocin signaling pathway, and MAPK signaling pathway are worthy of further exploration.

**Supplementary Information:**

The online version contains supplementary material available at 10.1186/s12864-021-07993-5.

## Background

Phosphorylation is one of the important posttranslational modifications of proteins; it is related to many activities of life, such as signal transduction, gene expression, cell cycle and cell apoptosis [1]. A phosphoric acid group with a strong negative charge is added to an amino acid of the protein, thereby changing its structure, activity and ability to interact with other molecules, affecting many biological processes. In eukaryotes, phosphorylation occurs primarily on serine, threonine and tyrosine [2].

Goat milk is becoming very popular with the improvement of people’s living conditions and their understanding of the unique nutritional and health value of goat milk. Therefore, improving the milk production and quality of dairy goats is the focus of our work. Dynamic changes in protein phosphorylation occur in goat mammary epithelial cells during the PL period and LL period [3, 4]. Previous studies have shown that protein phosphorylation regulates lactation in animals [5, 6]. Although phosphoproteome analysis was conducted on the oocytes of goats [7] and the heart and liver of mice [8, 9], phosphorylated proteomic analysis has not been performed in the PL and LL stages of dairy goat mammary tissues. The regulatory mechanism of phosphorylated proteins during lactation is not well known.

Therefore, in this experiment, differentially modified phosphorylation sites of phosphorylated proteins between PL and LL in goat mammary tissues were identified by tandem mass tag (TMT)/isobaric tags for relative and absolute quantitation (iTRAQ) labeling, high-performance liquid chromatography (HPLC) fractionation, affinity enrichment and liquid chromatography with tandem mass spectrometry (LC–MS/MS) analysis. A total of 1,938 (1,111 upregulated, 827 downregulated) differentially modified phosphorylation sites of 1,172 proteins were identified and analyzed with the Gene Ontology (GO) classification system and Kyoto Encyclopedia of Genes (KEGG) for differentially phosphorylated proteins.

The phosphorylation levels of ACACA (Ser80), EIF4EBP1 (Thr46), and IRS1 (Ser312) were increased, and that of JUN (Ser63) was decreased in PL compared with LL, and the data showed that the phosphoproteome results were reliable. The present study provides essential information to enhance our knowledge of the expression dynamics of phosphorylated proteins and phosphorylated sites during PL and LL, and it provides reference data for further studying the roles of protein phosphorylation during lactation in goat mammary tissues.

## Methods

### Mammary tissue collection

Guanzhong dairy goats were obtained from Longxian Goat Breeding Center, in Longxian County of Shaanxi Province, China. All procedures in our animal study were approved by the Animal Care and Use Committee of the Northwest A&F University (Yangling, China) (permit number: 17–347, data:2017-10-13). Mammary tissues were collected during the LL (280 days postpartum) and PL (90 days postpartum) periods from 18 healthy Guanzhong dairy goats (3 year olds) by surgery (9 mammary tissues in PL and 9 mammary tissues in LL). First, one side of the udder was wiped clean with alcohol, and then local anesthesia was applied to the tissue (Xilagesic 20 %, Calier, Barcelona, Spain). A 5–6 cm incision was made and the skin was pulled back using sterile forceps, exposing the mammary tissue. Samples (1 cm^2^) were taken using a sterile scalpel blade and forceps. Pressure was applied with sterile gauze to stop any bleeding. The incision was closed with 6 to 8 surgical staples (#89,063,337, Appose ULC Skin Stapler, 35 wide; Henry Schein Inc., Melville, NY). After that, the goat was kept in a clean place during the healing period, and the skin around the wound was sterilized with iodine tincture and 75 % alcoholregularly. All collected tissues were washed with RNase-free PBS, quickly frozen in liquid nitrogen and stored at -80 °C.

### Protein extraction

The mammary tissues epithelial tissues of dairy goats in PL and LL were grinded into cell powder in the presence of liquid nitrogen. The cell powder was transferred to a 5 mL centrifuge tube. Subsequently, the cell powder was added to four volumes of lysis buffer (8 M urea, 1 % Protease Inhibitor Cocktail, 2 nM EDTA), followed by thrice of ultrasonic treatment on ice with a high-intensity ultrasonic processor (Scientz, Ningbo, China). The remaining debris was removed by centrifugation at 12,000 g at 4 °C for 10 min. Finally, the supernatant was collected, and the protein concentration was quantified using the BCA kit (Scientz, Ningbo, China). Proteins were extracted from 18 mammary tissues of dairy goats that included nine mammary tissues in PL and nine mammary tissues in LL. Afterwards, proteins extracted from nine mammary tissues in PL were divided into three groups. Each group was made up of three parts of protein extracted from PL mammary tissues. The same method was used to construct the LL protein library.

### Trypsin digestion

The protein solution was reduced with 5 mM dithiothreitol for 30 min at 56 °C and alkylated with 11 mM iodoacetamide for 15 min at room temperature in the dark. Consequently, the protein solution was digested. The protein sample was diluted by adding 100 mM triethylammonium bicarbonate (TEAB). The concentration of urea in the solution should be less than 2 M. Finally, trypsin and protein were added at 1:50 mass ratio for the first digestion overnight and at 1:100 mass ratio for the second digestion for 4 h.

### TMT labelling and HPLC fractionation

The production was desalted by a Strata X C18 SPE column (Phenomenex, Torrance, USA) after trypsin digestion and vacuum drying. Afterwards, peptides were reconstituted in 0.5 M TEAB and processed using a TMT kit (Scientz, Ningbo, China). Peptides were labelled using TMT for quantitation; labelling was performed as previously described [10]. One unit of TMT reagent was thawed and reconstituted in acetonitrile. The peptide mixtures were incubated for 2 h at room temperature, pooled, desalted and dried by vacuum centrifugation. The tryptic peptides were divided into segments using a Thermo Betasil C18 column (5 μm particles, 10 mm ID, 250 mm length) by high pH reversed-phase HPLC. In summary, peptides were separated into 60 fractions after more than 60 min of gradient treatment with 8–32 % acetonitrile (pH 9.0). Subsequently, the peptides were combined into eight fractions and dried by vacuum centrifuging. Reactions were quenched using 8 µL of 11 % lysine following the manufacturer’s recommendations. Finally, 10 % trifluoroacetic acid (TFA, Sigma–Aldrich) was added into the peptide solutions. The volume ratio of the peptide solution to TFA was 1:10, and the mixture was pooled, desalted and dried by vacuum centrifugation.

### Affinity enrichment

The mixtures were first incubated with immobilised metal affinity chromatography (IMAC) microsphere suspensions with vibration in loading buffer (50 % acetonitrile/6 % TFA). Phosphopeptide-rich IMAC microspheres were collected by centrifugation. Afterwards, the supernatant was removed. The IMAC microspheres were washed with 50 % acetonitrile/6 % TFA and 30 % acetonitrile/0.1 % TFA to remove nonspecifically adsorbed peptides. To elute the enriched phosphopeptides from the IMAC microspheres, an elution buffer containing 10 % NH_4_OH was added, and the enriched phosphopeptides were eluted with vibration. The supernatant containing phosphopeptides was collected for LC–MS/MS analysis after lyophilisation.

### LC–MS/MS analysis

The tryptic peptides were dissolved in 0.1 % formic acid (solvent A), directly loaded onto a homemade reversed-phase analytical column (15 cm length, 75 μm i.d.). The gradient consisted of solvent B (0.1 % formic acid in 98 % acetonitrile), which increased from 6 to 23 % over 26 min, 23–35 % in 8 min and climbing to 80 % in 3 min, followed by holding at 80 % for the last 3 min, all at a constant flow rate of 400 nL/min on an EASY-Nlc 1,000 ultra-performance liquid chromatography (UPLC) system.

Peptides were characterised using tandem mass spectrometry (MS/MS) in Q Exactive™ Plus (Thermo) coupled online to the UPLC after the peptides were subjected to NSI source. The electrospray voltage applied was 2.0 Kv. The m/z scan was 350 to 1,800 for full scan, and intact peptides were detected in the Orbitrap at a resolution of 70,000. Using a normalization collision energy setting of 28, the fragments were detected in the Orbitrap at a resolution of 17,500 for the selected MS/MS of peptides. A data-dependent procedure was alternated between one MS scan followed by 20 MS/MS scans with a 15.0 s dynamic exclusion. The automatic gain control was set at 5E4. The fixed first mass was set at 100 m/z.

### Database search

Maxquant search engine (V.1.5.2.8) was used to process the resulting MS/MS data. The UniProt Ovis aries (27,472 sequences) concatenated with a decoy database that was used for the tandem mass spectra search. The search parameters were as follows: 6-plex TMT, the minimum length of the peptides was set at 7 amino acid residues and the maximum modification number of the peptides was set at 5; fixed modification (carbamidomethylation of cysteine residues); variable modification (protein N-term acetylation); and oxidation of methionine residues and phosphorylation of serine, threonine and tyrosine residues, thereby allowing two missing cleavages by cleavage enzyme trypsin/P. The mass error was set at 20 ppm in the first search and 5 ppm in the primary search, and the fragment ions of mass tolerance were set at 0.02 Da. The minimum score for modified peptides was set at > 40, and a false discovery rate was set at < 1 %.

### Bioinformatics analysis

Gene Ontology (GO) annotation data were obtained from UniProt GoA database. Proteins were divided into three categories by GO annotation, namely, a biological process, cellular compartment and molecular function. The GO with a corrected *P* value < 0.05 was considered significant. InterProScan (http://www.ebi.ac.uk/interpro/) was used to detect the enrichment of functional domains of differentially expressed proteins compared with all identified proteins. Protein domains with a corrected *P* value < 0.05 were considered significant. The KEGG database (https://www.genome.jp/kegg/tool/map_pathway2.html) was used to identify enriched pathways [11, 12]. The pathway with a corrected *P* value < 0.05 was considered significant. Based on the KEGG website, these pathways were classified into hierarchical categories. Fisher’s exact test was used to examine the enrichment of the differentially phosphorylation protein against all identified phosphorylation proteins. Wolfpsort, a subcellular localisation predication wolf, was used to predict subcellular localisation. Soft motif-x was used to analyse the sequence model constituted with amino acids in specific positions of modify-21-mers (10 amino acids upstream and downstream of the site) in all protein sequences. In addition, all database protein sequences were used as background database parameter, as well as other parameters with default.

### Enrichment-based clustering

Hierarchical clustering was based on the functional classification of different proteins. Categories that were at least enriched in one of the clusters with *P* < 0.05 were filtered after collating all the categories and their *P* values. This filtered *P* value matrix was transformed by the function *x* = − log10 (*P* value). Finally, these *x* values were *z*-transformed for each functional category. These *z* scores were then clustered by one-way hierarchical clustering (Euclidean distance and average linkage clustering) in Genesis. Cluster membership was visualised by a heat map using the “heatmap.2” function from the “gplots” R-package.

### Western blot

Approximately 25 µg of protein extracted from the goat mammary gland epithelial tissues was separated by SDS-PAGE and then transferred onto polyvinylidene difluoride membranes (PVDF, Merck Millipore, MA, USA). The membranes were immersed in 10 % nonfat milk powder in Tris-buffered saline containing 0.1 % Tween 20 (pH 7.6) for 2.5 h at room temperature. The membrane was incubated with the corresponding primary antibodies overnight at 4 °C (Table 1). In the next step, the membrane was incubated with the suitable HRP-conjugated secondary antibodies against mouse and rabbit at 4 °C for 2 h. The proteins were visualized using ECL prime western blotting detection reagent (Amersham, GE Healthcare Lifesciences, Sweden) by gel documentation system (Biospectrum 410, UVP). Proteins were quantified by the Quantity One program (Bio-Rad, California, USA).

**Table 1:** The antibody used in the present study

Name	Manufacturer	Product number
β-Actin	Beyotime, Shanghai, China	AA128
JUN	Signalway Antibody, College Park, USA	GTX82231
p-JUN (Ser63)	Signalway Antibody, College Park, USA	CY5418
ACACA	Signalway Antibody, College Park, USA	CY5854
p-ACACA(Ser80)	Signalway Antibody, College Park, USA	CY5168
EIF4EBP1	Signalway Antibody, College Park, USA	CY5277
p-EIF4EBP1(Thr46)	Signalway Antibody, College Park, USA	A0208
IRS1	Abcam, UK	A0216
p-IRS1(Ser312)	Signalway Antibody, College Park, USA	11,143
HRP-labeled Goat Anti-Rabbit IgG (H + L)	Beyotime, Shanghai, China	A0208
HRP-labeled Goat Anti-Mouse IgG (H + L)	Beyotime, Shanghai, China	A0216

### Statistics

In this study, the reporter ion intensity of the modified peptide was used to calculate the modification site quantitation. First, the average quantitation of each sample was calculated in multiple replicates, and then the ratio of the average quantitation between the two samples was calculated. The ratio is used as the relative quantitation between the two samples. The next step is to calculate the significant *p* value of differential modification between the two samples. First, the relative quantitative values of each sample are taken as log2 transform (so that the data conformed to the normal distribution), and the *p* value was calculated by the two-sample two-tailed T-test method. A *p* value < 0.05 and a modification ratio > fold change were regarded as upregulation. A *p* value < 0.05 and protein ratio < 1/fold change was regarded as downregulation. Each experiment was repeated at least three times. All of the data were processed by SPSS 19.0 (Beijing, China). The western blot results are shown as the means ± SE (standard error), and the differences were compared by the two-sample two-tailed T-test method (**P < 0.01, *P < 0.05).

## Results

### Sample repeatability test and mass spectrometry quality control

**Fig. 1:**
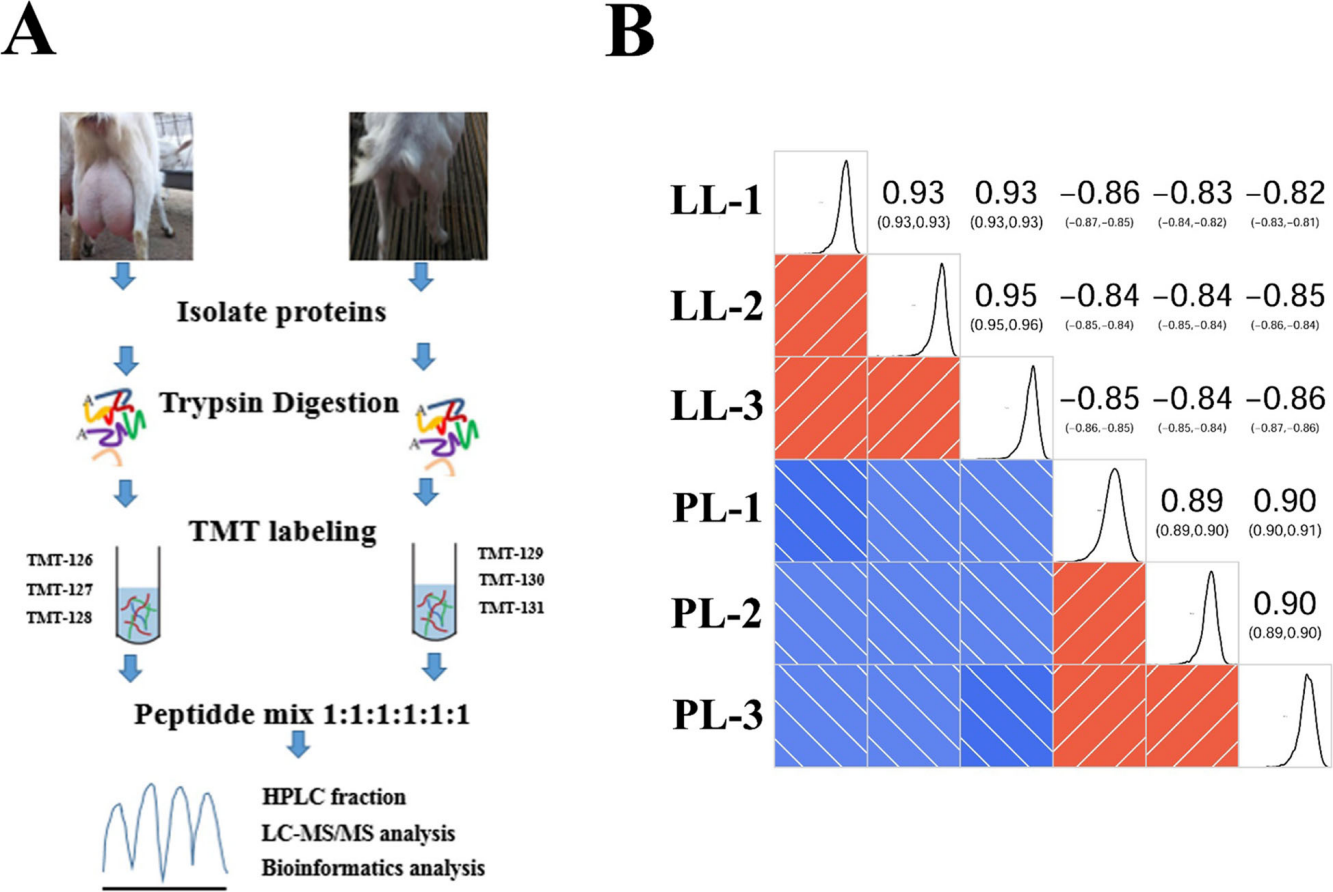
Experimental strategy for quantitative proteome analysis and quality control validation of MS data. **A** The workflow of the study; proteins were extracted in three biological replicates for each sample group. **B** Pearson’s Correlation Coefficient of protein quantitation

Differentially modified phosphorylation sites and phosphorylated proteins from the PL and LL of goats were analyzed by LC–MS/MS and TMT. The workflow of the present study is shown in Fig. 1a. The results of the sample repeatability test are shown in Fig. 1b. Figure 1b is a heatmap drawn by calculating Pearson’s correlation coefficient between two pairs of samples. Pearson’s correlation coefficient showed that the reproducibility of the sample was good. The mass error was detected at 0 as the center axis and was concentrated in a range of less than 10 ppm. The length of most peptide segments was between 8 and 20 amino acid residues, which indicated that the preparation of the sample was successful in our study (Figure S1).

### Analysis of differentially phosphorylated proteins

**Fig. 2:**
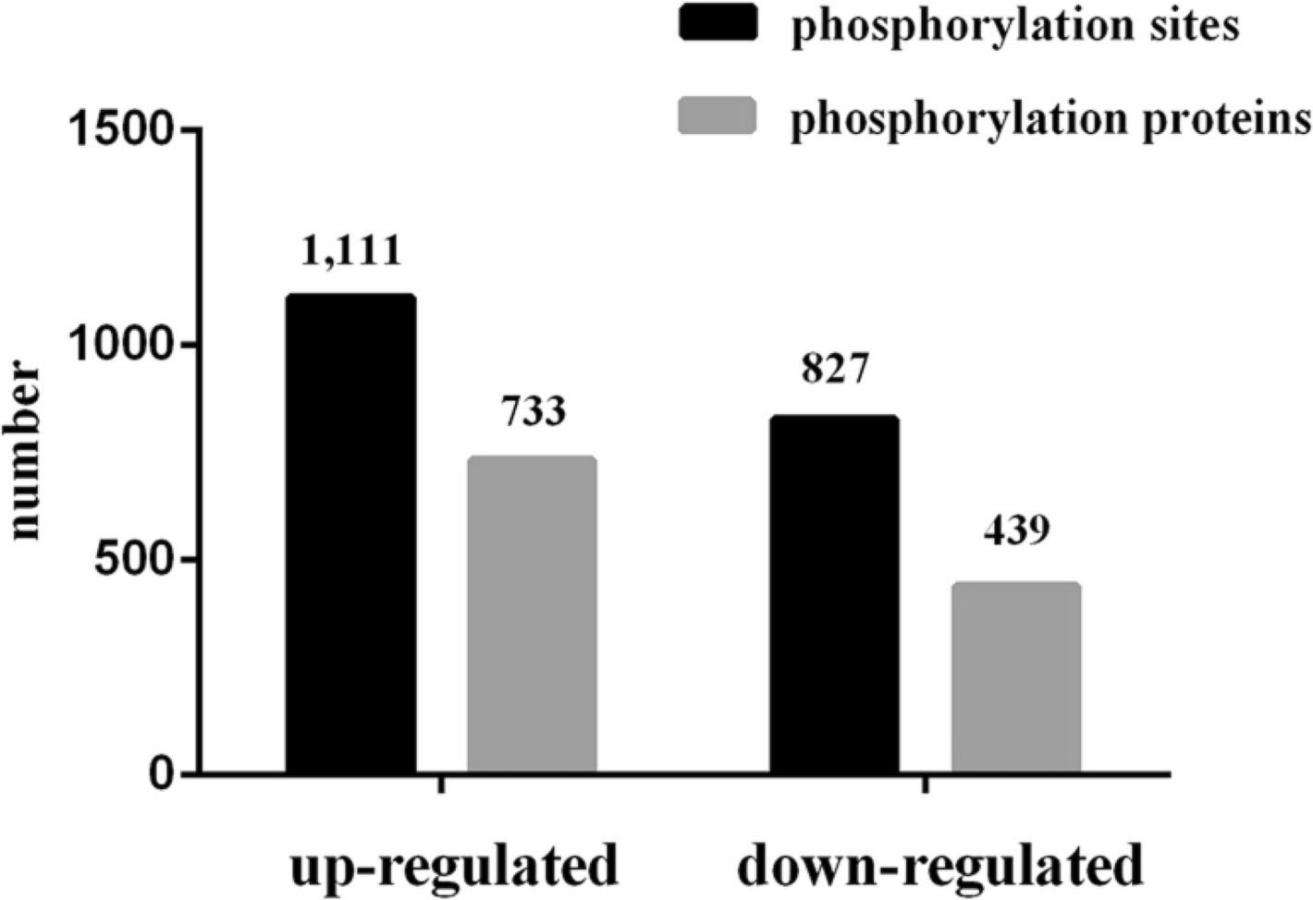
The numbers of differentially modified phosphorylation sites and phosphorylated proteins

A quantitative study of differentially modified phosphorylation sites was performed in this experiment by an integrated approach involving LC–MS/MS and TMT labeling. All samples were prepared in triplicate. There were 6,979 phosphorylated sites distributed on 2,608 proteins. In addition, 5,901 phosphorylated sites distributed on 2,454 proteins were quantified. In the classified groups, the variation of the difference expressed was more than 1.5 times and less than 0.67 times the change criterion of significant upregulation or significant downregulated (*P* values < 0.05). A total of 1,938 (1,111 up-regulated and 827 down-regulated) differentially modified phosphorylation sites of 1,172 proteins were changed (Fig. 2; Table S1). In addition, we found for the first time that there were multiple differentially modified phosphorylation sites between PL and LL, including FASN (modified phosphorylation sites: 2120, 2212, 2174, 2178, 2206, 63, 2126, 2420 and 324), ACACA (29, 80, 1251 and 25), mTOR (1261), PRKAA (377 and 395), IRS1 (629, 312, 1138 and 1070), RPS6KB (374 and 394), EIF4EBP1 (65) and TSC2 (1719, 1327 and 918).

To identify the phosphorylated proteins, the phosphorylated proteins and their differentially modified phosphorylation sites were annotated in detail by GO and KEGG pathways (Table S2).

**Fig. 3:**
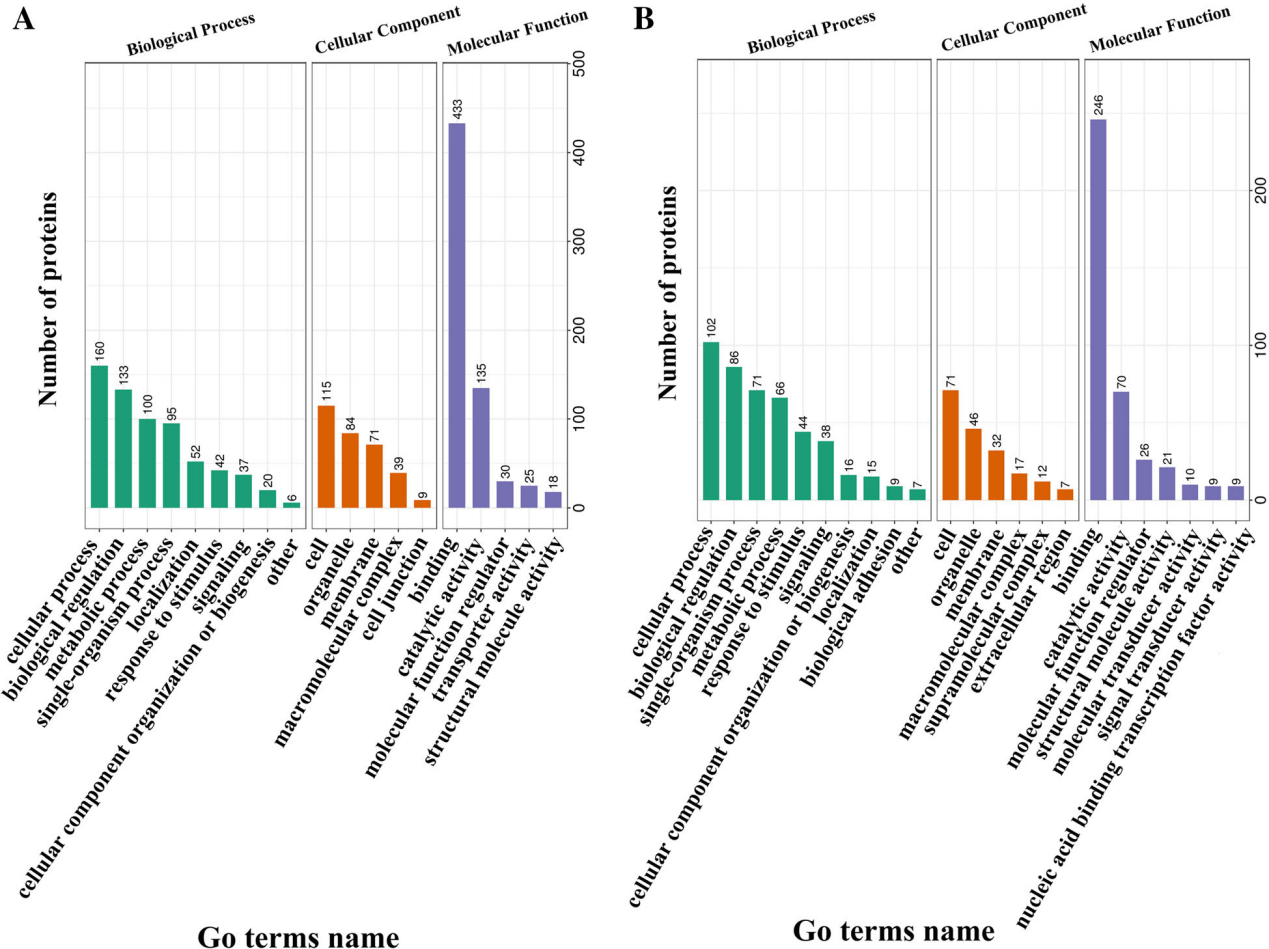
Distribution of the up-regulated phosphorylated proteins (**A**: biological process, cellular component, molecular function) and down-regulated phosphorylated proteins (**B**: biological process, cellular component, molecular function) with GO annotation

A total of 2,454 proteins with quantitative information were classified by GO annotation based on three categories, namely, biological process, cellular component and molecular function. The results showed that ‘metabolic process’, ‘biological regulation’ and ‘catalytic activity’ were significantly enriched. ‘Metabolic process’ contained 133 upregulated phosphorylated proteins and 66 downregulated phosphorylated proteins, ‘biological regulation’ contained 100 upregulated phosphorylated proteins and 86 downregulated phosphorylated proteins, and ‘catalytic activity’ contained 135 upregulated phosphorylated proteins and 70 downregulated phosphorylated proteins (Fig. 3, Table S3).

### Functional enrichment of the differentially phosphorylated proteins

**Fig. 4:**
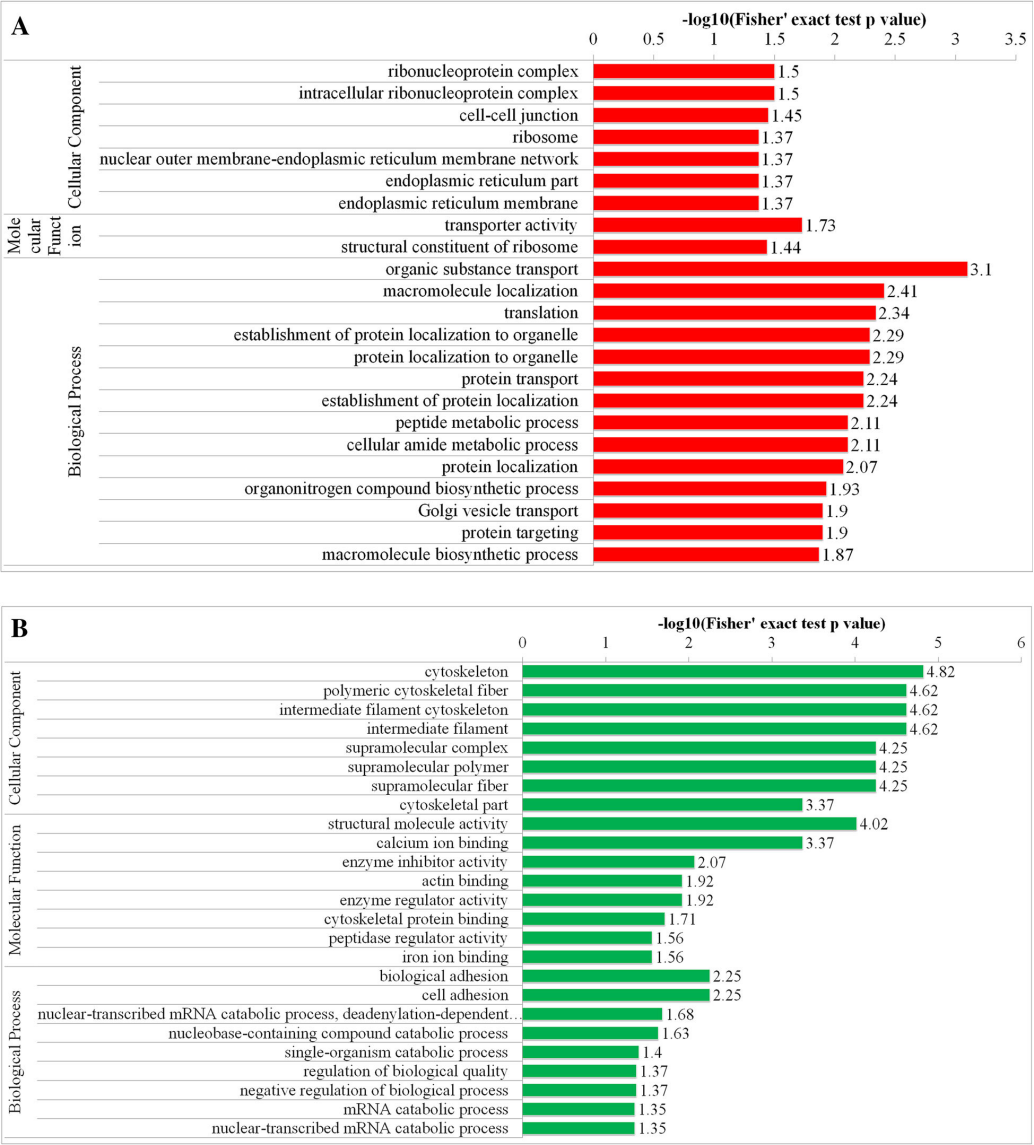
Remarkably enriched GO terms of the up-regulated phosphorylated proteins (**A**) and down-regulated phosphorylated proteins (**B**) concerning cellular component, molecular function and biological process

Proteins that contained differentially modified phosphorylation sites were analyzed by GO enrichment analysis. From the analysis of the results, up- and downregulated phosphorylated site corresponding proteins were primarily enriched to translation, transportation and energy metabolism, such as ‘translation’, ‘organic substance transport’, ‘Golgi vesicle transport’ and, ‘transporter activity’ (Fig. 4).

**Fig. 5:**
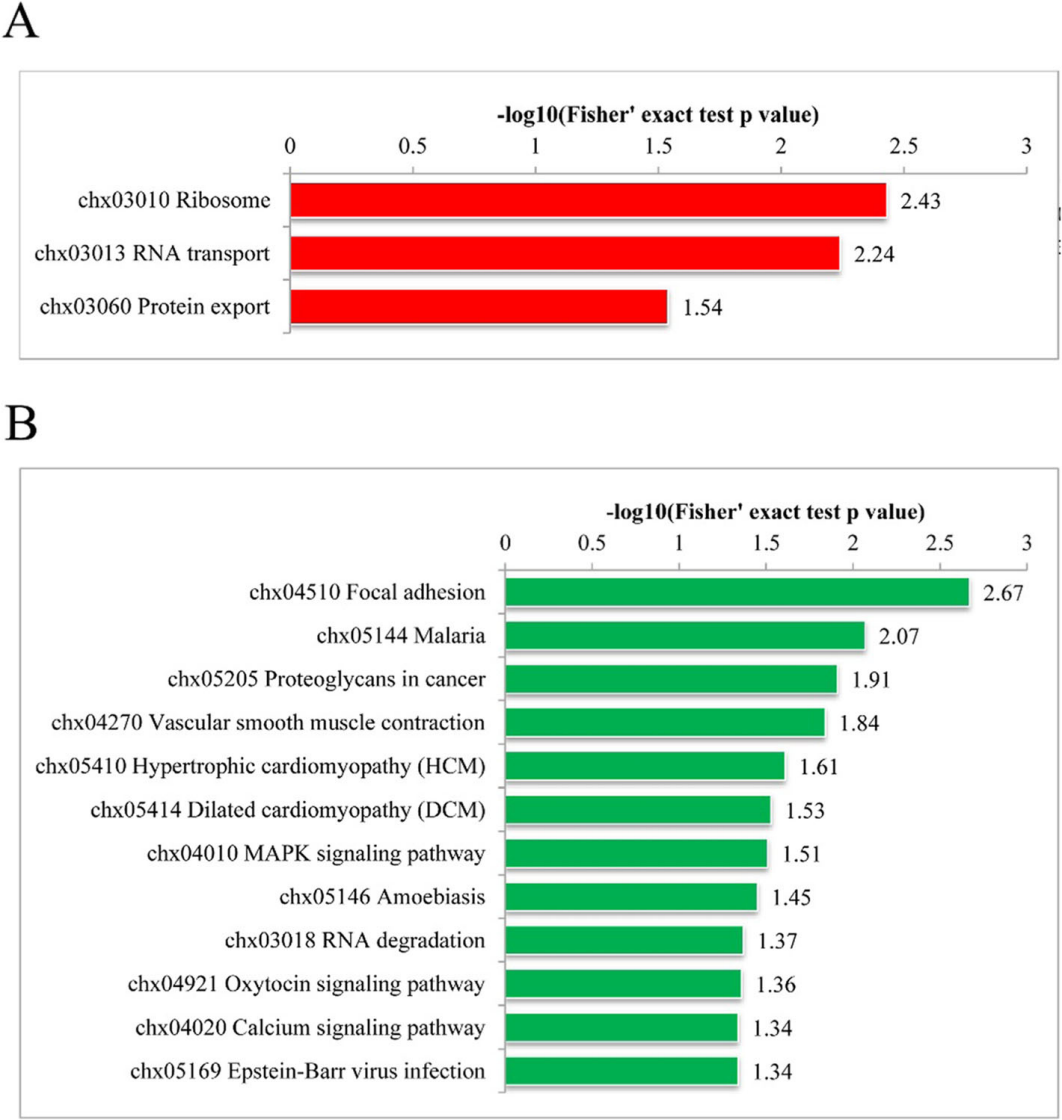
KEGG enrichment analysis of the up-regulated phosphorylated proteins (**A**) and down-regulated phosphorylated proteins (**B**)

KEGG enrichment analysis revealed that the proteins that contained differentially phosphorylated sites were remarkably enriched in the ‘MAPK signaling pathway’, ‘Oxytocin signaling pathway’ and ‘Calcium signaling pathway’ which are highly correlated with lactation (Fig. 5).

### Validation of changes in the phosphorylated protein levels by western blot

**Fig. 6:**
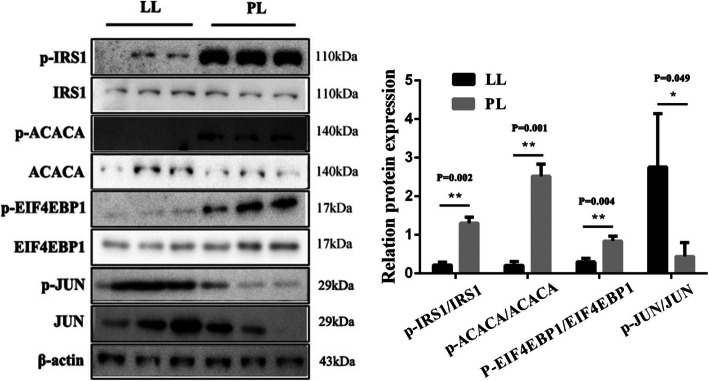
Protein levels of p-IRS1(full-length blots/gels are presented in Figure S2), IRS1(full-length blots/gels are presented in Figure S3), *p*-ACACA(full-length blots/gels are presented in Figure S4), ACACA(full-length blots/gels are presented in Figure S5), *p*-EIF4EBP1(full-length blots/gels are presented in Figure S6), EIF4EBP1(full-length blots/gels are presented in Figure S7), *p*-JUN(full-length blots/gels are presented in Figure S8), JUN(full-length blots/gels are presented in Figure S9) in PL and LL were detected by western blot. Full-length blots/gels of β-actin are presented in Figure S10

Phosphorylation levels in PL and LL of ACACA (Ser80), EIF4EBP1 (Thr46), IRS1 (Ser312) and JUN (Ser63) were tested by western blot. The results showed that the phosphorylation levels of ACACA, EIF4EBP1 and IRS1 increased and JUN decreased in PL compared with LL (Fig. 6).

## Discussion

Protein phosphorylation regulates many cellular processes in eukaryotic cells by posttranslational modification [13]. Protein phosphorylation reveals the effects of temperature on wheat leaves and spikelets [14], and the importance of protein phosphorylation has been demonstrated for different chloroplast functions [15]. Protein phosphorylation has an important role in sperm quality [16] and an important connection with lactation [5, 17]. The level of phosphorylation constantly changes throughout the development of mammary tissues and during the different stages of lactation [3]. Therefore, in this study, differentially phosphorylated proteins in PL and LL were detected by LC–MS/MS and TMT. These results will contribute to the elucidation of the mechanism underlying the regulation of lactation in goat mammary tissues by phosphorylation.

The production of milk increases after parturition and subsequently reaches its peak [18]. These phenomena are dependent on a series of factors, including somatotropin, insulin and functional protein. Nevertheless, the effect of protein phosphorylation on goat mammary tissues during lactation remains unclear. Therefore, differentially phosphorylated proteins were explored in goat mammary tissues. A total of 1,938 differentially modified phosphorylation sites of 1,172 proteins were found between PL and LL. The western blot results of the phosphorylation levels of ACACA, EIF4EBP1, IRS1 and JUN showed that the phosphoproteome results were reliable, and these results are consistent with other studies [19, 20]. The mTOR pathway is a key regulatory pathway in lactation. PRKAA, IRS1, RPS6KB, EIF4EBP1, TSC2, FASN and ACACA are located in the mTOR pathway. Previous studies showed that activation of mTOR pathway can enhance the synthesis of lactose and triglycerides [21, 22], and researchers has shown that APOC3, FASN and ACACA proteins are highly correlated with the decomposition and utilization of fat [23–25]. Moreover, the inactivation of PLIN4 could decrease fat accumulation [26].

Insulin regulates glucose uptake and metabolism by binding to an insulin receptor [27], and insulin receptors (IRS1 and IRS2) are necessary for insulinfunction. In a previous study, Cryer, A et al. [28] demonstrated that activation of ACACA could promote fatty acid synthesis in adipocytes. Barber, M.C et al. [29] showed that lipid synthetic capacity during early lactation is associated with activity of FASN.

Zhang, M. C. et al. [30] demonstrated that an upregulated phosphorylation level of mTOR (2448) stimulates casein synthesis in dairy cows. Burgos, S. A. et al. [31] found that phosphorylated TSC2 (1462) and EIF4EBP1 (37/46) could regulate protein synthesis in bovine mammary epithelial cells.

However, the differentially modified phosphorylation sites of the proteins identified in this study (FASN (modified phosphorylation sites: 2120, 2212, 2174, 2178, 2206, 63, 2126, 2420 and 324), ACACA (29, 80, 1251 and 25), mTOR (1261), PRKAA (377 and 395), IRS1 (629, 312, 1138 and 1070), RPS6KB (374 and 394), EIF4EBP1 (65) and TSC2 (1719, 1327 and 918)) have never been explored. Therefore, we speculate that the changes in these phosphorylated sites may be strongly related to lactation.

The energy metabolism of mammary tissues increases remarkably during lactation [32]. GO enrichment showed several differentially phosphorylated proteins enriched in biological processes, which were related to energy metabolism, material transport and protein translation. These results provide an overview of dynamic changes during lactation. A series of signaling pathways were enriched in PL compared with LL, such as the ‘calcium signaling pathway’ (chx04020), ‘oxytocin signaling pathway’ (chx04921) and ‘MAPK signaling pathway’ (chx04010). Among them, the three signaling pathways of ‘calcium signaling pathway’, ‘oxytocin signaling pathway’, and ‘MAPK signaling pathway’ are highly related to lactation [33–37]. Therefore, we speculate that the changes in these phosphorylated sites in calcium signaling pathway, oxytocin signaling pathway, and MAPK signaling pathway may be related to lactation. These phosphorylated sites will become the research focus of our future work. This study provided a better understanding of the function of phosphorylated proteins in goat mammary gland epithelial tissues and provided a reference for future research.

## Conclusions

The present study was the first to conduct sequence phosphorylated proteomics in the mammary tissues of dairy goats, and the findings of this study markedly increased the current understanding of posttranslational phosphorylation of protein events that occur during the progression of lactation. The western blot results of the phosphorylation levels of ACACA, EIF4EBP1, IRS1 and JUN showed that the phosphoproteome results were reliable. Multiple differentially modified phosphorylation sites of FASN, ACACA, mTOR, PRKAA, IRS1, RPS6KB, EIF4EBP1, TSC2, and JUN and proteins of the calcium signalling pathway, oxytocin signaling pathway, and MAPK signaling pathway might have potential research value in regulating goat lactation. We will study the function of these proteins and their differentially modified phosphorylation sites during lactation in the future. Therefore, the present study provides valuable reference information for further study of the molecular mechanism of lactation in goats.

## Supplementary Information

**Additional file 1: Figure S1.** Length distribution of all identified peptides.

**Additional file 2: Figure S2.** Full-length blot of p-IRS1 protein expression level.

**Additional file 3: Figure S3.** Full-length blot of IRS1 protein expression level.

**Additional file 4: Figure S4.** Full-length blot of p-ACACA protein expression level.

**Additional file 5: Figure S5.** Full-length blot of ACACA protein expression level.

**Additional file 6: Figure S6.** Full-length blot of p-EIF4EBP1 protein expression level.

**Additional file 7: Figure S7.** Full-length blot of EIF4EBP1 protein expression level.

**Additional file 8: Figure S8.** Full-length blot of p-JUN protein expression level.

**Additional file 9: Figure S9.** Full-length blot of JUN protein expression level.

**Additional file 10: Figure S10.** Full-length blot of β-actin protein expression level.

**Additional file 11: Table S1.**  Annotation information of all differentially phosphorylated proteins and modified modification sites.

**Additional file 12: Table S2.** Detailed information of all differentially phosphorylated proteins and modified modification sites.

**Additional file 13: Table S3.**  Functional categorisation of differentially phosphorylated proteins.

## Data Availability

The mass spectrometry proteomics data have been deposited to the ProteomeXchange Consortium via the PRIDE partner repository with the dataset identifier PXD026437 (http://www.ebi.ac.uk/pride).

## Abbreviations

FASN: Fatty acid synthase
ACACA: Acetyl-CoA carboxylase 1 isoform X3
mTOR: Serine/threonine-protein kinase mTOR
RPS6KB: Ribosomal protein S6 kinase beta-1 isoform X1
IRS1: Insulin receptor substrate 1
PRKAA: 5′-AMP-activated protein kinase catalytic subunit alpha-2
RPS6KB: Ribosomal protein S6 kinase beta-1 isoform X1
EIF4EBP1: Eukaryotic translation initiation factor 4E-binding protein 1
TSC2: Tuberin
JUN: Transcription factor AP-1

## References

[1] Humphrey SJ, Yang G, Yang P, Fazakerley DJ, Stockli J, Yang JY, James DE (2013). Dynamic adipocyte phosphoproteome reveals that Akt directly regulates mTORC2. Cell Metab.

[2] Wu HY, Liao PC (2008). Analysis of protein phosphorylation using mass spectrometry. Chang Gung Med J.

[3] Foncea R, Varela S, Sapag-Hagar M, Lavandero S (1995). Changes in protein kinase C activity, subcellular distribution and protein phosphorylation during the lactogenic cycle in the rat mammary tissue. Res Commun Mol Pathol Pharmacol.

[4] Jones FE, Welte T, Fu XY, Stern DF (1999). ErbB4 signaling in the mammary gland is required for lobuloalveolar development and Stat5 activation during lactation. J Cell Biol.

[5] Lin Y, Duan X, Lv H, Yang Y, Liu Y, Gao X, Hou X (2018). The effects of L-type amino acid transporter 1 on milk protein synthesis in mammary glands of dairy cows. J Dairy Sci.

[6] Li H, Liu X, Wang Z, Lin X, Yan Z, Cao Q, Zhao M, Shi K (2017). MEN1/Menin regulates milk protein synthesis through mTOR signaling in mammary epithelial cells. Sci Rep.

[7] Gall L, Le Gal F, De Smedt V (1993). Protein phosphorylation patterns during in vitro maturation of the goat oocyte. Mol Reprod Dev.

[8] Chang YW, Chang YT, Wang Q, Lin JJ, Chen YJ, Chen CC (2013). Quantitative phosphoproteomic study of pressure-overloaded mouse heart reveals dynamin-related protein 1 as a modulator of cardiac hypertrophy. Mol Cell Proteomics.

[9] Wilson-Grady JT, Haas W, Gygi SP (2013). Quantitative comparison of the fasted and re-fed mouse liver phosphoproteomes using lower pH reductive dimethylation. Methods (San Diego, Calif).

[10] Plubell DL, Wilmarth PA, Zhao Y, Fenton AM, Minnier J, Reddy AP, Klimek J, Yang X, David LL, Pamir N (2017). Extended Multiplexing of Tandem Mass Tags (TMT) Labeling Reveals Age and High Fat Diet Specific Proteome Changes in Mouse Epididymal Adipose Tissue. Mol Cell Proteomics.

[11] Kanehisa M, Goto S (2000). KEGG: kyoto encyclopedia of genes and genomes. Nucleic Acids Res.

[12] Kanehisa M (2019). Toward understanding the origin and evolution of cellular organisms. Protein Sci.

[13] Preisinger C, von Kriegsheim A, Matallanas D, Kolch W (2008). Proteomics and phosphoproteomics for the mapping of cellular signalling networks. Proteomics.

[14] Vu LD, Zhu T, Verstraeten I, van de Cotte B, Gevaert K, De Smet I (2018). Temperature-induced changes in the wheat phosphoproteome reveal temperature-regulated interconversion of phosphoforms. J Exp Bot.

[15] Reiland S, Messerli G, Baerenfaller K, Gerrits B, Endler A, Grossmann J, Gruissem W, Baginsky S (2009). Large-scale Arabidopsis phosphoproteome profiling reveals novel chloroplast kinase substrates and phosphorylation networks. Plant Physiol.

[16] Shen ZQ, Shi B, Wang TR, Jiao J, Shang XJ, Wu QJ, Zhou YM, Cao TF, Du Q, Wang XX (2019). Characterization of the sperm proteome and reproductive outcomes with in vitro, fertilization after a reduction in male ejaculatory abstinence period. Mol Cell Proteomics.

[17] Liu J, Wang Y, Li D, Wang Y, Li M, Chen C, Fang X, Chen H, Zhang C. Milk protein synthesis is regulated by T1R1/T1R3, a G protein-coupled taste receptor, through the mTOR pathway in the mouse mammary gland. Mol Nutr Food Res. 2017;61(9):1601017-49. 10.1002/mnfr.201601017 28497545

[18] Wiltbank MC, Baez GM, Garcia-Guerra A, Toledo MZ, Monteiro PL, Melo LF, Ochoa JC, Santos JE, Sartori R (2016). Pivotal periods for pregnancy loss during the first trimester of gestation in lactating dairy cows. Theriogenology..

[19] Liu Y, Hou J, Zhang M, Seleh-Zo E, Wang J, Cao B, An X. circ-016910 sponges miR-574-5p to regulate cell physiology and milk synthesis via MAPK and PI3K/AKT-mTOR pathways in GMECs. J Cell Physiol. 2020;235(5):4198–216. 10.1002/jcp.29370 PMC7028128 31663119

[20] Chen K, Hou J, Song Y, Zhang X, Liu Y, Zhang G, Wen K, Ma H, Li G, Cao B (2018). Chi-miR-3031 regulates beta-casein via the PI3K/AKT-mTOR signaling pathway in goat mammary epithelial cells (GMECs). BMC Vet Res.

[21] Wang L, Lin Y, Bian Y, Liu L, Shao L, Lin L, Qu B, Zhao F, Gao X, Li Q (2014). Leucyl-tRNA synthetase regulates lactation and cell proliferation via mTOR signaling in dairy cow mammary epithelial cells. Int J Mol Sci.

[22] Zhu C, Wang L, Zhu J, Jiang Y, Du X, Duan Q, Yin H, Huang X, Song Y, Cao B et al. OGR1 negatively regulates β-casein and triglyceride synthesis and cell proliferation via the PI3K/AKT/mTOR signaling pathway in goat mammary epithelial cells. Anim Biotechnol. 2020:1–10. 10.1080/10495398.2020.1737099 32167419

[23] Russo GT, Meigs JB, Cupples LA, Demissie S, Otvos JD, Wilson PW, Lahoz C, Cucinotta D, Couture P, Mallory T (2001). Association of the Sst-I polymorphism at the APOC3 gene locus with variations in lipid levels, lipoprotein subclass profiles and coronary heart disease risk: the Framingham offspring study. Atherosclerosis.

[24] Travers MT, Cambot M, Kennedy HT, Lenoir GM, Barber MC, Joulin V (2005). Asymmetric expression of transcripts derived from the shared promoter between the divergently oriented ACACA and TADA2L genes. Genomics.

[25] Hao J, Zhu L, Zhao S, Liu S, Liu Q, Duan H (2011). PTEN ameliorates high glucose-induced lipid deposits through regulating SREBP-1/FASN/ACC pathway in renal proximal tubular cells. Exp Cell Res.

[26] Chen W, Chang B, Wu X, Li L, Sleeman M, Chan L (2013). Inactivation of Plin4 downregulates Plin5 and reduces cardiac lipid accumulation in mice. Am J Physiol Endocrinol Metab.

[27] Li Y, Soos TJ, Li X, Wu J, Degennaro M, Sun X, Littman DR, Birnbaum MJ, Polakiewicz RD (2004). Protein kinase C Theta inhibits insulin signaling by phosphorylating IRS1 at Ser(1101). J Biol Chem.

[28] Vernon RG, Clegg RA: Chap. 4 - The metabolism of white adipose tissue in vivo and in vitro. In: New Perspectives in Adipose Tissue. Edited by Cryer A, Van RLR. Oxford: Butterworth-Heinemann; 1985:65–86.

[29] Barber MC, Clegg RA, Travers MT, Vernon RG (1997). Lipid metabolism in the lactating mammary gland. Biochim Biophys Acta.

[30] Zhang MC, Zhao SG, Wang SS, Luo CC, Gao HN, Zheng N, Wang JQ (2018). d-Glucose and amino acid deficiency inhibits casein synthesis through JAK2/STAT5 and AMPK/mTOR signaling pathways in mammary epithelial cells of dairy cows. J Dairy Sci.

[31] Burgos SA, Cant JP (2010). IGF-1 stimulates protein synthesis by enhanced signaling through mTORC1 in bovine mammary epithelial cells. Domest Anim Endocrinol.

[32] Gross JJ, Schwarz FJ, Eder K, van Dorland HA, Bruckmaier RM (2013). Liver fat content and lipid metabolism in dairy cows during early lactation and during a mid-lactation feed restriction. J Dairy Sci.

[33] Zhang R, Ma H, Gao Y, Wu Y, Qiao Y, Geng A, Cai C, Han Y, Zeng YA, Liu X (2018). Th-POK regulates mammary gland lactation through mTOR-SREBP pathway. PLoS Genetics.

[34] Gimpl G, Fahrenholz F (2001). The oxytocin receptor system: structure, function, and regulation. Physiol Rev.

[35] Elabd C, Cousin W, Upadhyayula P, Chen RY, Chooljian MS, Li J, Kung S, Jiang KP, Conboy IM (2014). Oxytocin is an age-specific circulating hormone that is necessary for muscle maintenance and regeneration. Nat Commun.

[36] Grinman D, Athonvarungkul D, Wysolmerski J, Jeong J (2020). Calcium metabolism and breast cancer: echoes of lactation?. Curr Opin Endocrine Metab Res.

[37] Hou J, An X, Song Y, Cao B, Yang H, Zhang Z, Shen W, Li Y (2017). Detection and comparison of microRNAs in the caprine mammary gland tissues of colostrum and common milk stages. BMC Genetics.

