# Supplementary figures and images for: Exploration of the lactation function of protein phosphorylation sites in goat mammary tissues by phosphoproteome analysis
Source: BMC Genomics. 2021 Sep 28;22:703. doi: 10.1186/s12864-021-07993-5 (PMC8479986; doi:10.1186/s12864-021-07993-5)

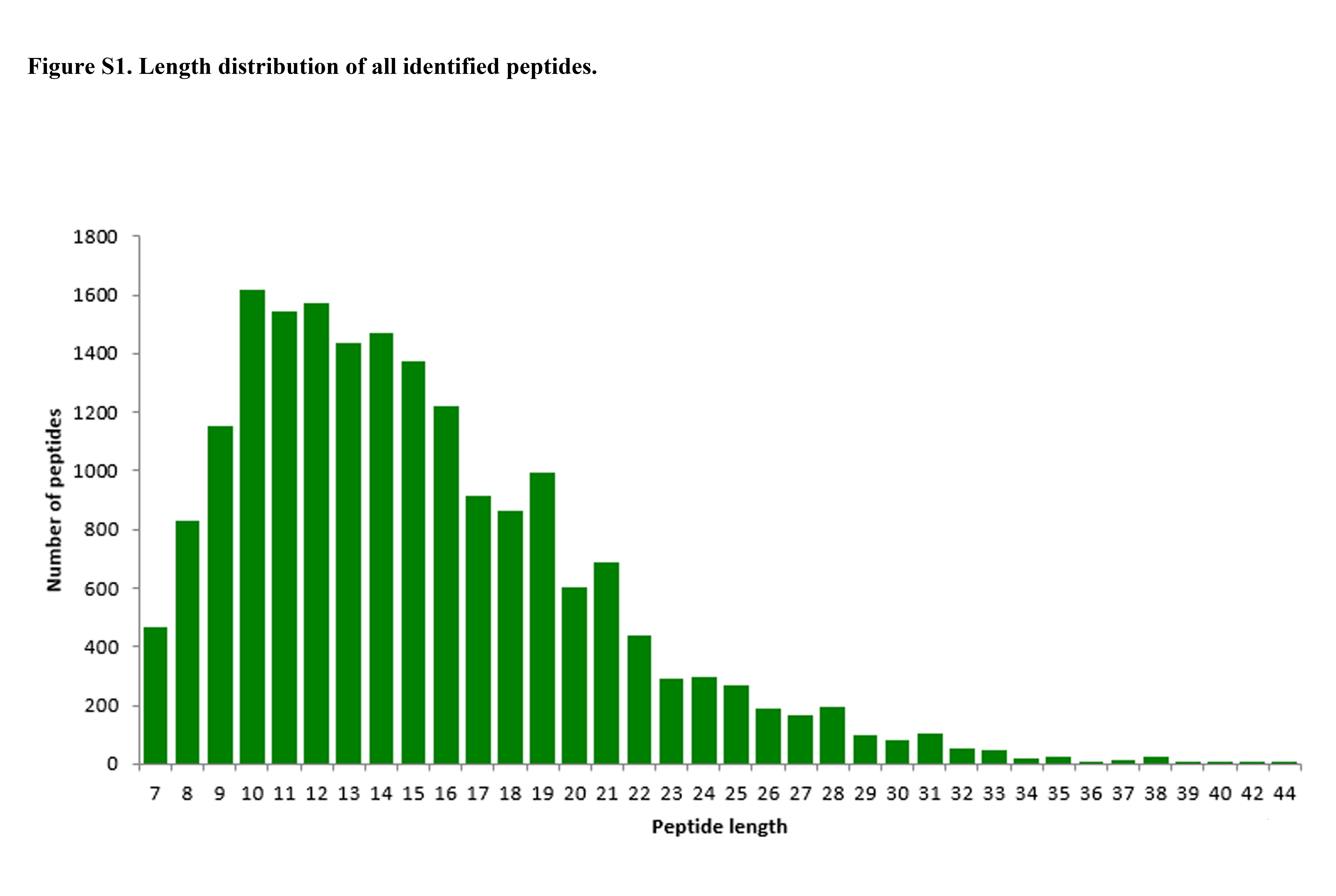

Supplement: Supplementary file 1 — Additional file 1: Figure S1. Length distribution of all identified peptides. [file 12864_2021_7993_MOESM1_ESM.jpg]

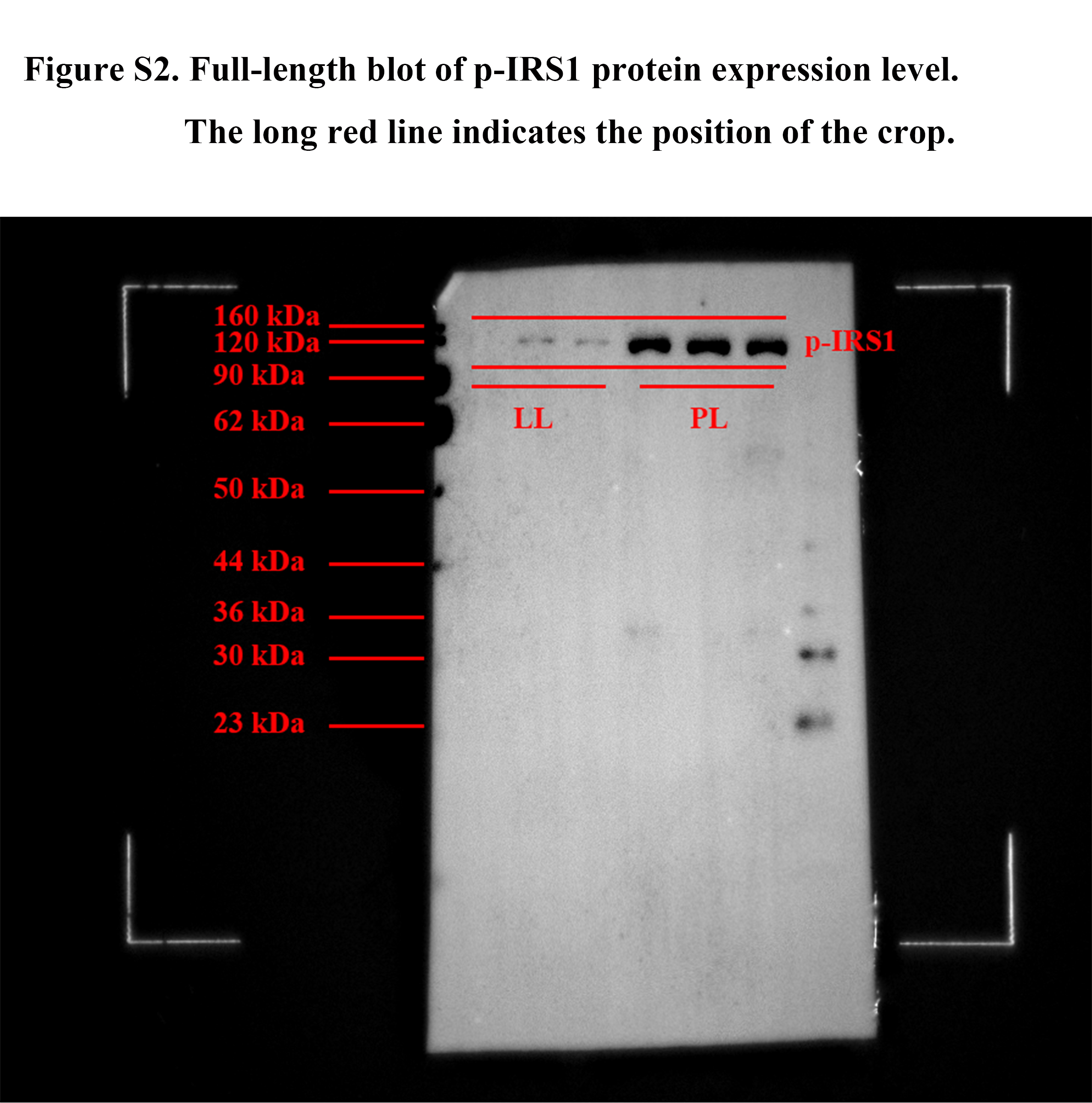

Supplement: Supplementary file 2 — Additional file 2: Figure S2. Full-length blot of p-IRS1 protein expression level. [file 12864_2021_7993_MOESM2_ESM.jpg]

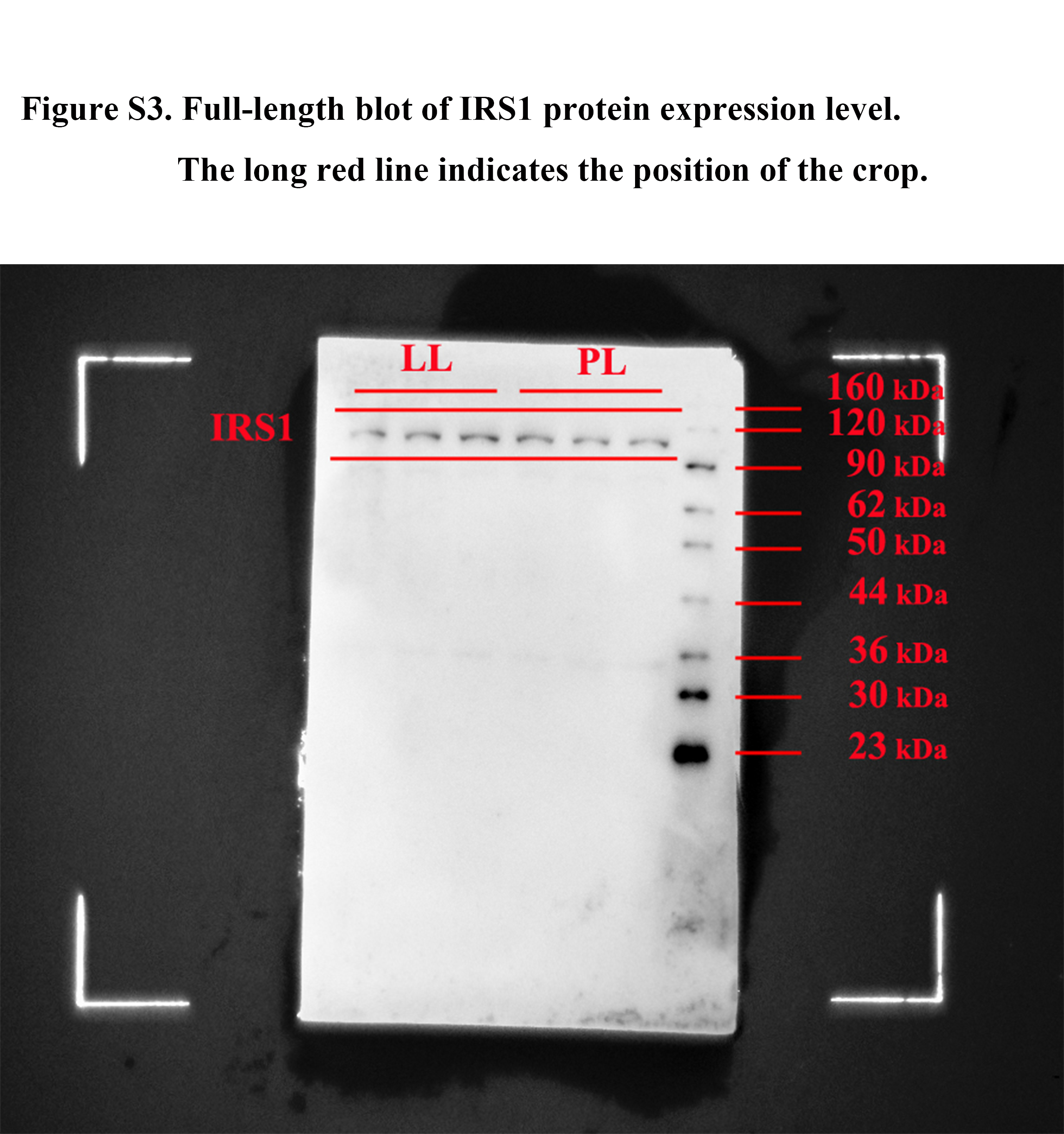

Supplement: Supplementary file 3 — Additional file 3: Figure S3. Full-length blot of IRS1 protein expression level. [file 12864_2021_7993_MOESM3_ESM.jpg]

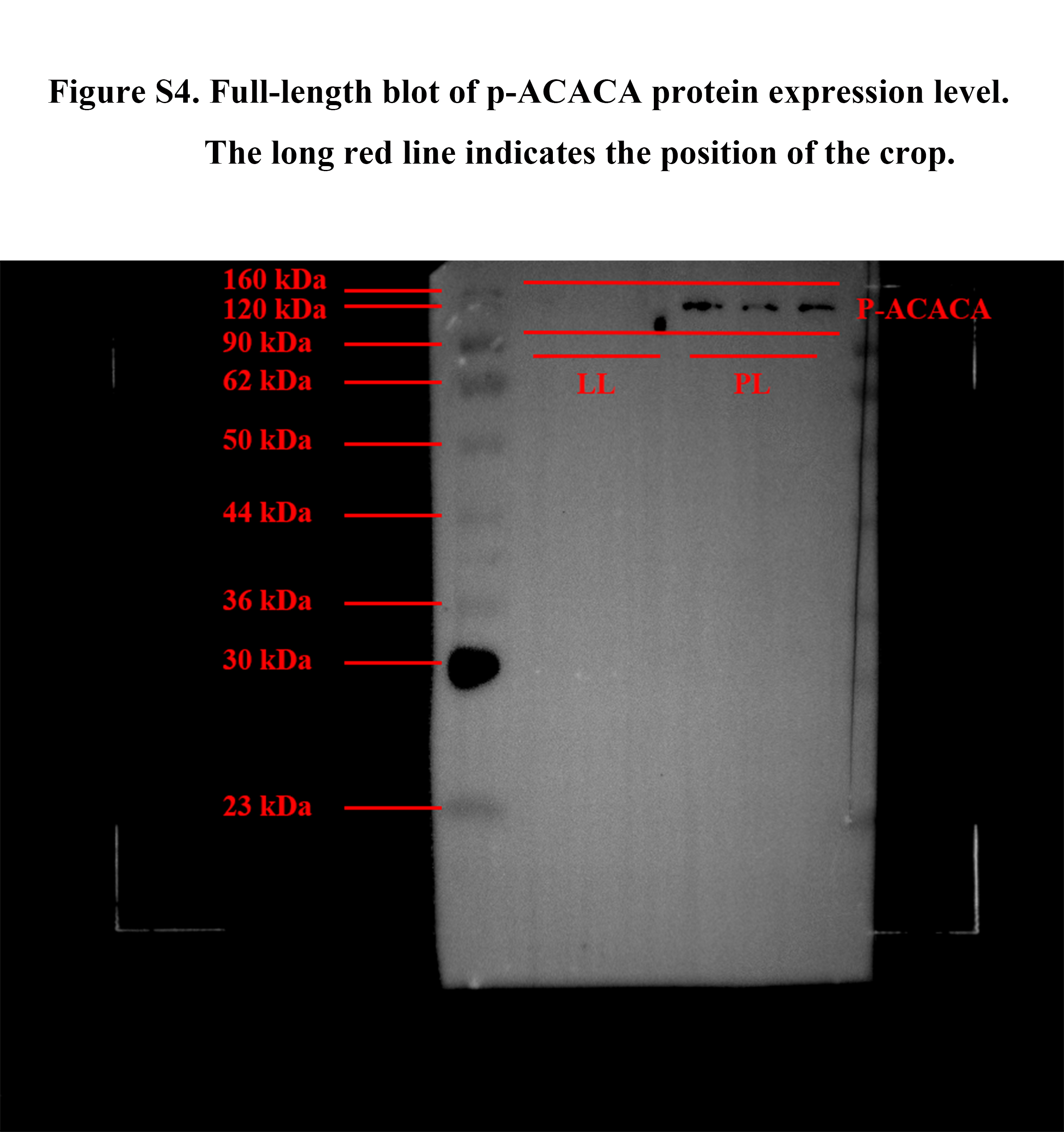

Supplement: Supplementary file 4 — Additional file 4: Figure S4. Full-length blot of p-ACACA protein expression level. [file 12864_2021_7993_MOESM4_ESM.jpg]

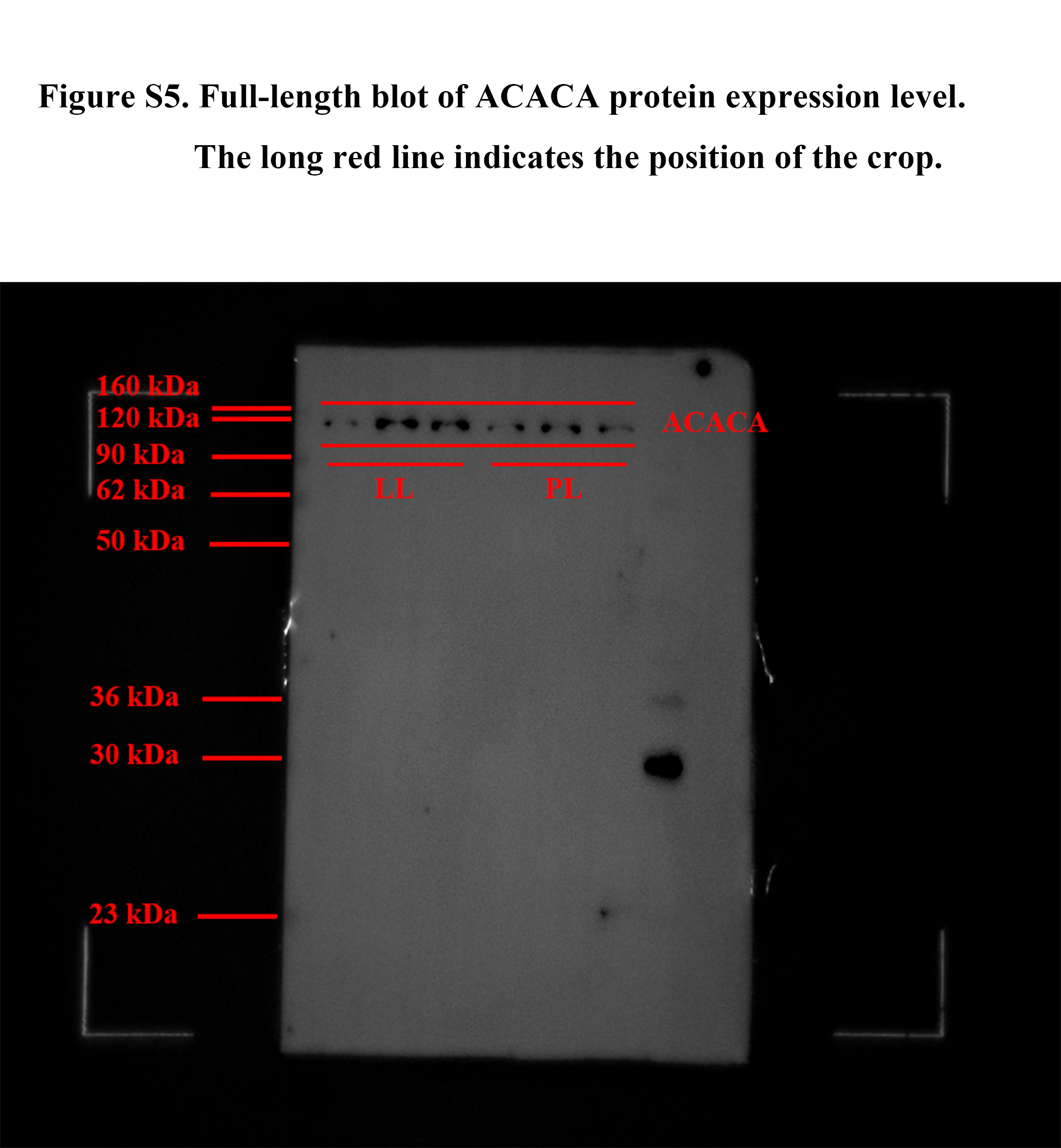

Supplement: Supplementary file 5 — Additional file 5: Figure S5. Full-length blot of ACACA protein expression level. [file 12864_2021_7993_MOESM5_ESM.jpg]

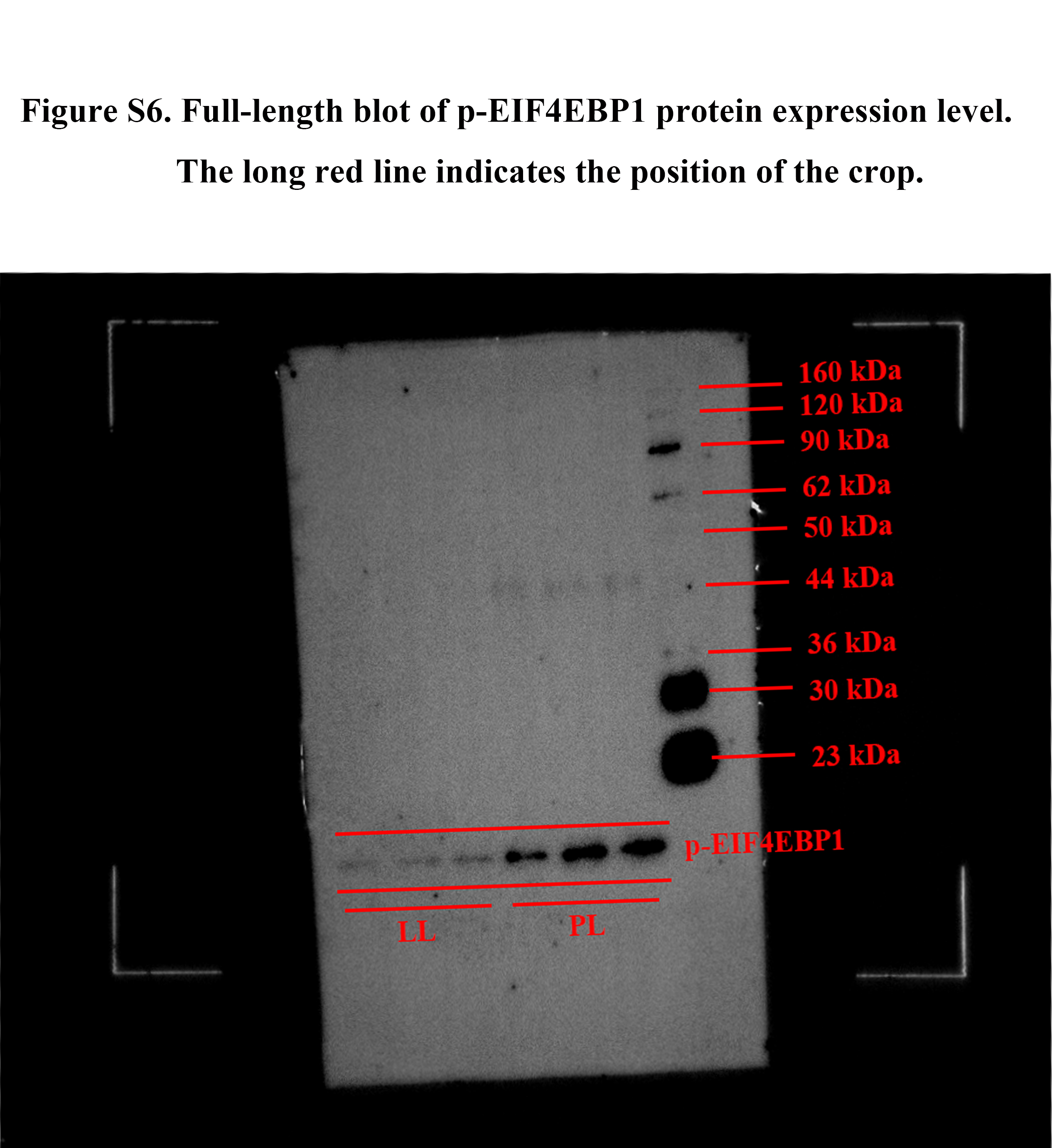

Supplement: Supplementary file 6 — Additional file 6: Figure S6. Full-length blot of p-EIF4EBP1 protein expression level. [file 12864_2021_7993_MOESM6_ESM.jpg]

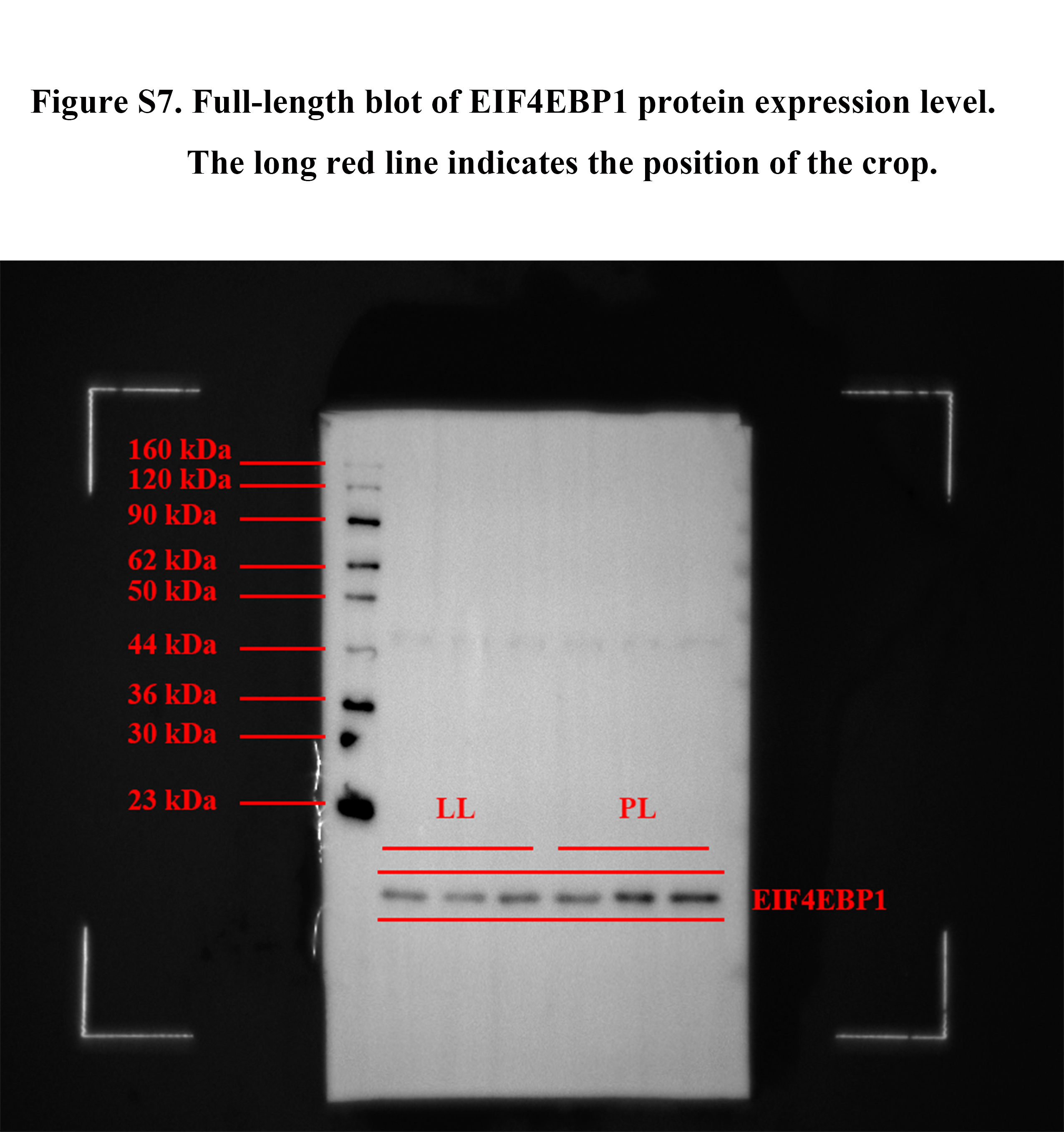

Supplement: Supplementary file 7 — Additional file 7: Figure S7. Full-length blot of EIF4EBP1 protein expression level. [file 12864_2021_7993_MOESM7_ESM.jpg]

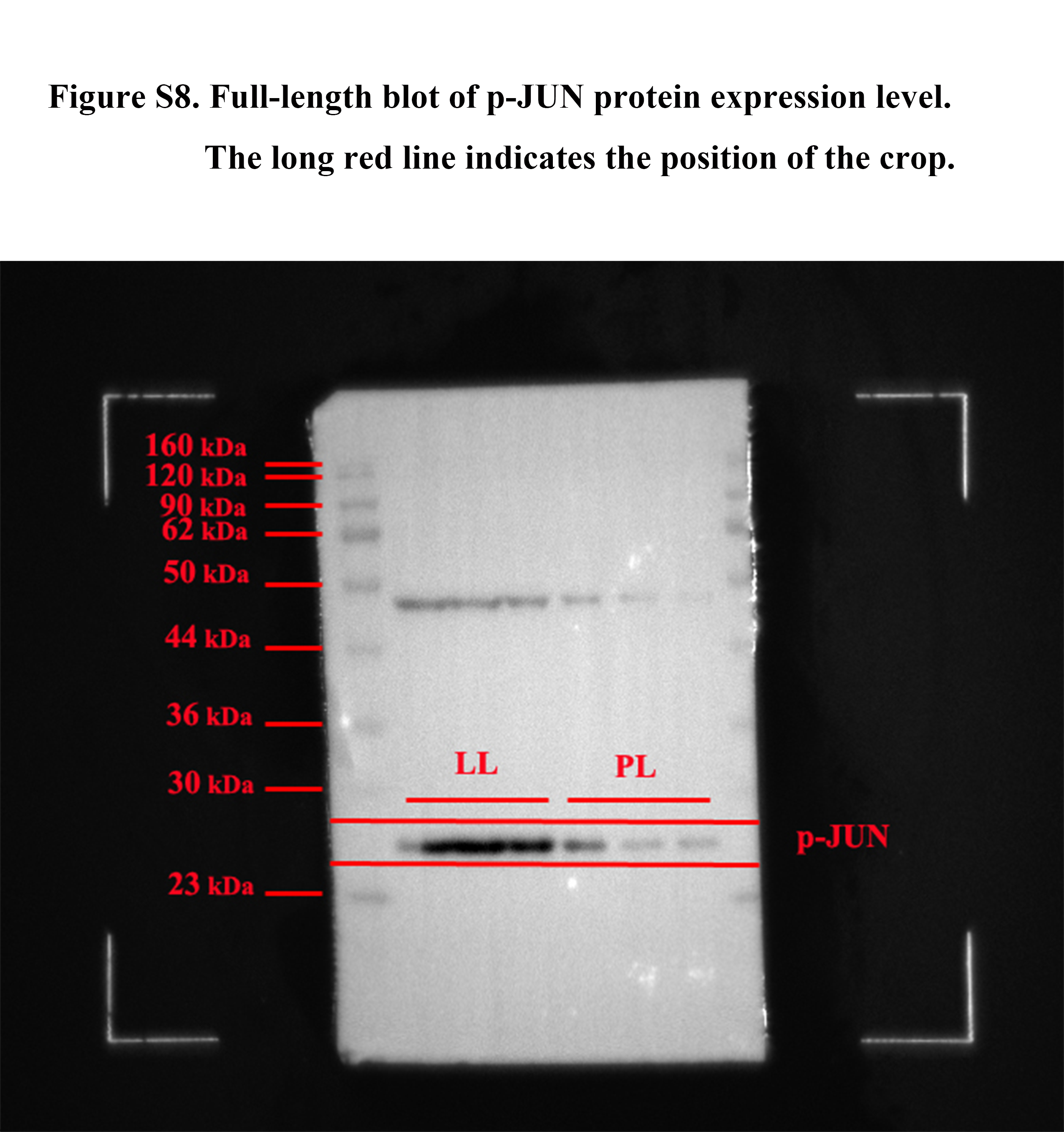

Supplement: Supplementary file 8 — Additional file 8: Figure S8. Full-length blot of p-JUN protein expression level. [file 12864_2021_7993_MOESM8_ESM.jpg]

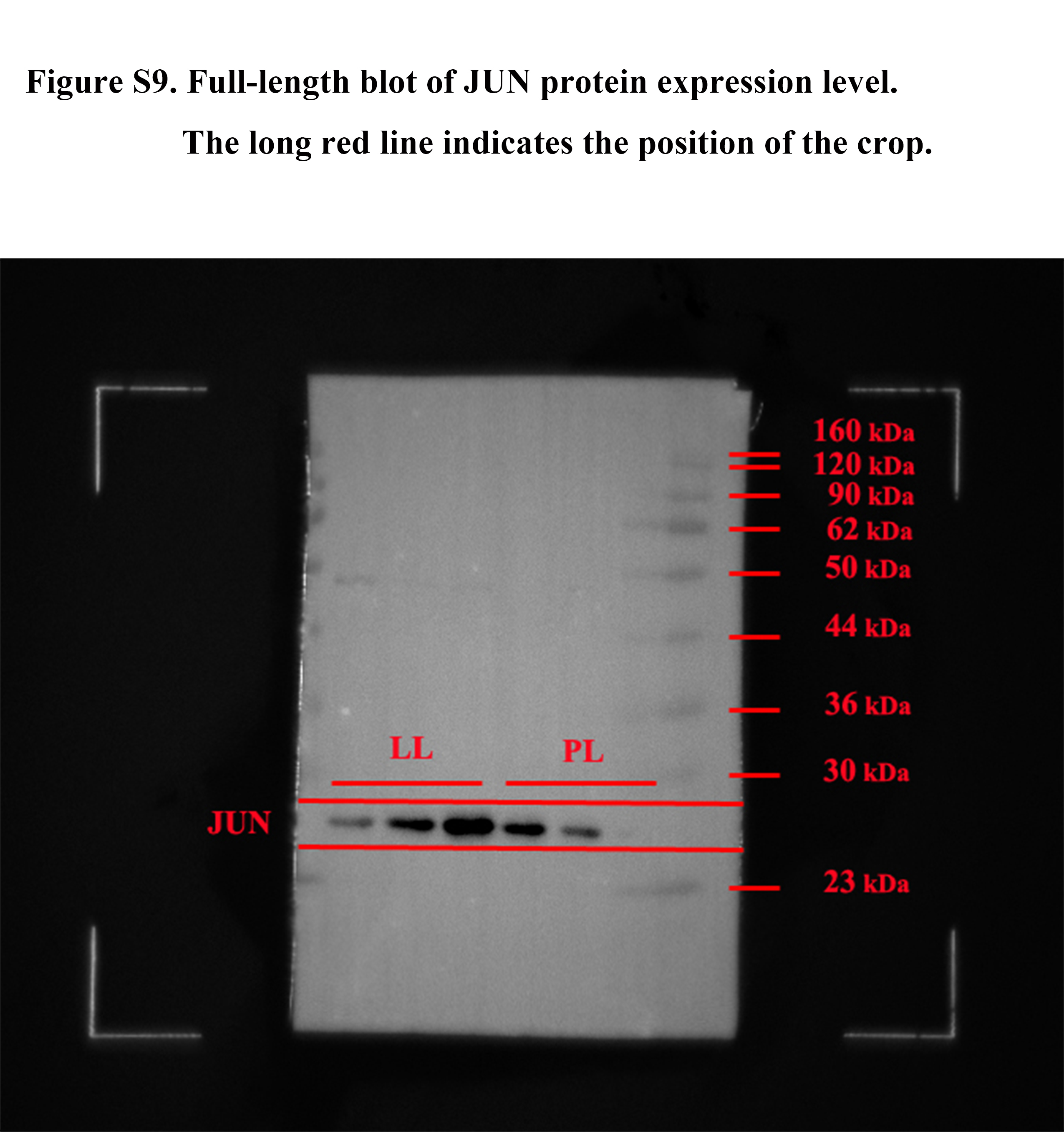

Supplement: Supplementary file 9 — Additional file 9: Figure S9. Full-length blot of JUN protein expression level. [file 12864_2021_7993_MOESM9_ESM.jpg]

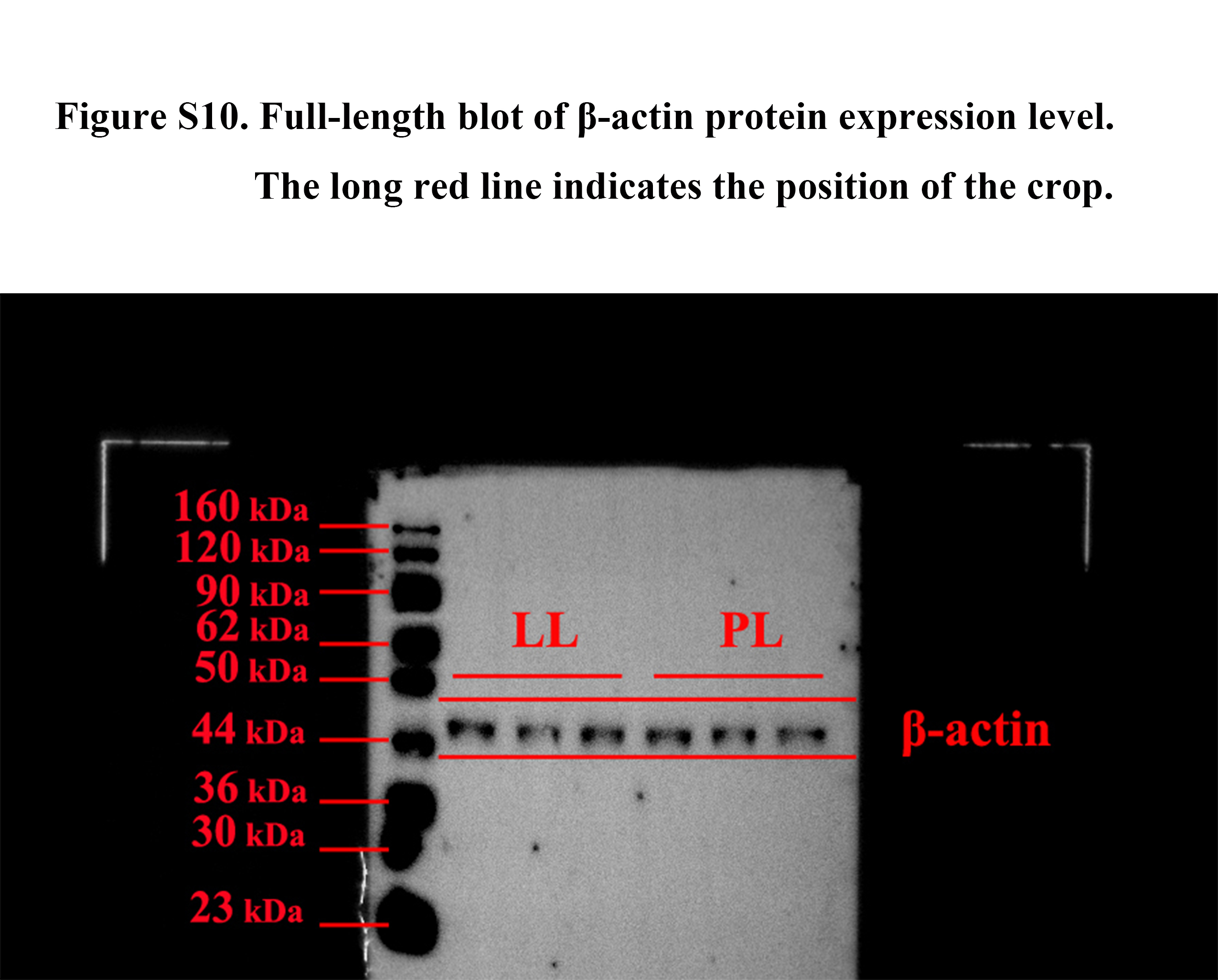

Supplement: Supplementary file 10 — Additional file 10: Figure S10. Full-length blot of β-actin protein expression level. [file 12864_2021_7993_MOESM10_ESM.jpg]
